# Supplementary material for: Correlation between clinical presentations, radiological findings and high risk histopathological features of primary enucleated eyes with advanced retinoblastoma at Queen Sirikit National Institute of Child Health: 5 years result
Source: PLoS One. 2022 Jul 20;17(7):e0270362. doi: 10.1371/journal.pone.0270362 (PMC9299328; doi:10.1371/journal.pone.0270362)
Supplement: Attachment — Submitted filename: Response to reviewers.docx [file pone.0270362.s002.docx]

**Response to reviewers’ comments**
**Reviewer #1:** Although there are many previous similar reports , which have described the high risk histopathological features after enucleation and yielded more or less similar results and conclusions, your study analyzed added the analysis of the correlation with radiological findings and clinical presentation.

1/3 of your included patients (11 out of 33) did CT ONLY as the imaging modality prior to enucleation. Apart from the fact you already mentioned in your introduction that the high dose of ionizing radiation used in CT should be avoided and carries a high risk in hereditary cases, MRI is definitely the scan of choice for detection of ON, choroid &/or scleral involvement. So you relied on a relatively less accurate test in 33% of your patients for the analysis of the radiological findings and its correlation with HP and clinical findings.

Response: The explanation of CT scan was under discussion “Most of our patients came from rural areas in Thailand and were admitted for investigation and enucleation in a single visit, so CT may be performed in some patients.”

The "Introduction" section is redundant and would be better summarized.

Response: Removed the sentence “The International Intraocular Retinoblastoma Classification (IIRC) classifies retinoblastoma as A–E, depending on tumor size, location, and presence of vitreous seeding and/or retinal detachment.” following reviewer 1 suggestion.

The English in the manuscript is not of publication quality and require major improvement and proof-reading.

Response: English proof was done by editage website.

**Reviewer #2:** The manuscript has been drafted with great efforts. I appreciate the hard-work put in by the team.
1. As there was an association seen between age and high-risk factors in the study, it will be interesting to have the p-value for the same as a part of the abstract too.

Response: Added P-value to abstract.

2. The conclusion section of the abstract has a blend of results and conclusion. It will be great if the authors could segregate the same.
Response: Moved the sentence “Unifocal intraocular mass with necrosis was the most common histopathological finding.” and “High-risk features were found in 45% of primary enucleated eye.” from the conclusion to the result section.

3. I think it will be a better idea to include the word "advanced retinoblastoma" as a part of the title too.

Response: Added the word “advanced retinoblastoma to the title”

4. The introduction has been written very well with appropriate information for the readers.

Response: Removed the sentence “The International Intraocular Retinoblastoma Classification (IIRC) classifies retinoblastoma as A–E, depending on tumor size, location, and presence of vitreous seeding and/or retinal detachment.” following reviewer 1 suggestion.

5. Line 135-136 - Advanced retinoblastoma term followed by grouping, shall cover the spectrum neatly. Kindly reconsider the terms used here. Or are all the groups included? It is slightly unclear here.

Response: According to ICRB classification, after clinical diagnosis and staging of the disease to group D or group E retinoblastoma. The globe salvage regimen or primary enucleation were provided as the options.

6. Line 137 - Was it a consecutive enrollment? If yes, please mention the same.

Response: Added “All thirty-three primary enucleated eyes were enrolled in this study.”

7. Line 143 - Was the chemotherapy given monthly or 2-monthly? Kindly highlight.

Response: After enucleation, all bilateral patients received systemic chemotherapy monthly and closed follow-up fundus examination every 2 months.

8. Line 145 - Was it just high IOP or NVG?

Response: The intraocular pressure were measure only in some patients, all the patients with proptosis revealed high intraocular pressure. The neovascular glaucoma was not noted in the record.

9. Table 2 - Inclusion of high IOP along with proptosis seems a little inaccurate. Best to cover NVG separately.

Response: The intraocular pressure were measure only in some patients, all the patients with proptosis revealed high intraocular pressure. The neovascular glaucoma was not noted in the record.

10. Line 153 - Please mention the number. As all the cases weren't group E.

Response: Rechecked the data, all the patients were group E retinoblastoma in one eye. According to ICRB classification, after clinical diagnosis and staging of the disease to group E retinoblastoma. Line 153 was not change.

11. Table 3 - It will be interesting if the authors could include the histopathological risk factors in the table and compare with the radiological findings. A reflection of the same gives a higher impact. It has been partly covered in table 6, but it will be best if combined while commenting on all the variables.

Response: Change table 6, added choroidal involvement and anterior chamber invasion. And added the discussion “Even though, the MRI revealed 100% specificity and 100% positive predictive value for choroidal invasion and anterior segment involvement, the sample size of this group is too small for making the conclusion.”

12. Line 190 - Highlighting again that proptosis and glaucoma are best if individually assessed and commented upon.

Response: The intraocular pressure were measure only in some patients, all the patients with proptosis revealed high intraocular pressure. The neovascular glaucoma was not noted in the record.

13. Kindly keep the percentage format symmetrical throughout the manuscript i.e. rounded to one decimal or 2 decimals or none.

Response: Keep the percentage format symmetrical throughout the manuscript rounded to none.

14. Line 232 - 233 - Kindly cite the reference here.

Response: Added reference [11].

15. Line 247 - A brief comment on the conclusion of the study is recommended.

Response: Added conclusion.
